# Anti-Tumor Activity and Mechanism of Silibinin Based on Network Pharmacology and Experimental Verification

**DOI:** 10.3390/molecules29081901

**Published:** 2024-04-22

**Authors:** Peihai Li, Dexu Wang, Xueliang Yang, Changyu Liu, Xiaobin Li, Xuanming Zhang, Kechun Liu, Yun Zhang, Mengqi Zhang, Changyun Wang, Rongchun Wang

**Affiliations:** 1 Engineering Research Center of Zebrafish Models for Human Diseases and Drug Screening of Shandong Province, Biology Institute, Qilu University of Technology (Shandong Academy of Sciences), Jinan 250103, China; liph@sdas.org (P.L.); dexuwangbio@163.com (D.W.);; 2 Key Laboratory of Novel Food Resources Processing, Ministry of Agriculture and Rural Affairs, Key Laboratory of Agro-Products Processing Technology of Shandong Province, Institute of Agro-Food Science and Technology, Shandong Academy of Agricultural Sciences, Jinan 250100, China; 3 Key Laboratory of Marine Drugs, The Ministry of Education of China, School of Medicine and Pharmacy, Ocean University of China, Qingdao 266003, China

**Keywords:** silibinin, anti-tumor, network pharmacology, adenoid cystic carcinoma, Western blot analysis

## Abstract

Silibinin is a flavonoid compound extracted from the seeds of *Silybum marianum* (L.) Gaertn. It has the functions of liver protection, blood-lipid reduction and anti-tumor effects. However, the potential molecular mechanism of silibinin against tumors is still unknown. This study aimed to assess the anti-tumor effects of silibinin in adenoid cystic carcinoma (ACC2) cells and Balb/c nude mice, and explore its potential mechanism based on network pharmacology prediction and experimental verification. A total of 347 targets interacting with silibinin were collected, and 75 targets related to the tumor growth process for silibinin were filtrated. Based on the PPI analysis, CASP3, SRC, ESR1, JAK2, PRKACA, HSPA8 and CAT showed stronger interactions with other factors and may be the key targets of silibinin for treating tumors. The predicted target proteins according to network pharmacology were verified using Western blot analysis in ACC2 cells and Balb/c nude mice. In the pharmacological experiment, silibinin was revealed to significantly inhibit viability, proliferation, migration and induce the apoptosis of ACC2 cells in vitro, as well as inhibit the growth and development of tumor tissue in vivo. Western blot analysis showed that silibinin affected the expression of proteins associated with cell proliferation, migration and apoptosis, such as MMP3, JNK, PPARα and JAK. The possible molecular mechanism involved in cancer pathways, PI3K-Akt signaling pathway and viral carcinogenesis pathway via the inhibition of CASP3, MMP3, SRC, MAPK10 and CDK6 and the activation of PPARα and JAK. Overall, our results provided insight into the pharmacological mechanisms of silibinin in the treatment of tumors. These results offer a support for the anti-tumor uses of silibinin.

## 1. Introduction

At present, tumors have become the second leading cause of global mortality following cardiovascular diseases, and the number of patients is steadily increasing year by year [1]. According to statistics, in 2018, there were about 18.1 million new cases of cancer worldwide, and about 9.6 million deaths from cancer, which indicates a serious threat to human life. Therefore, the search for highly effective and low-toxicity drugs for the treatment of cancer has become an important public health problem [2].

Traditional Chinese Medicine (TCM) has become an effective method to prevent and cure tumors because of its unique advantages and minimal side effects [3]. The flavonoids are widely found in various plant components of Chinese herbal medicines such as flowers, leaves and fruits. It has many pharmacological activities, such as anti-proliferative, anti-oxidative and anti-inflammatory activity and the ability to lower blood sugar levels; therefore, it can be applied to the clinical treatment of tumors, cardiovascular disease, diabetes and other diseases [4,5]. The TCM herb, *Silybum marianum* (L.) Gaertn (also known as Shuifeiji in Chinese) from the Compositae family, has been used for treating liver diseases, lowering blood lipids, anti-tumor activity, etc. [6,7,8,9,10]. Silibinin is a biologically active flavonoid ingredient extracted from the seeds of *S. marianum* (L.) Gaertn. It has the functions of protecting the liver, anti-tumor effects, diabetes prevention, lowering of blood lipid content and anti-oxidation effects [11,12]. Studies have shown that silibinin has a good inhibitory effect on a variety of tumors, especially liver tumors [11,13,14,15]. However, the molecular anti-tumor mechanism of silibinin is still unclear.

Network pharmacology based on gene, protein and disease database information has become an emerging and effective strategy to observe drug action at the organ and organism levels and investigate the pharmacological mechanism of TCMs and their active ingredients [16,17]. This method is used to predict compound–proteins/genes–disease relationships from a network perspective relating to drugs, bioactivity and diseases, and can be applied in different fields such as pharmacological mechanistic studies, drug development and targeted exploration [18,19]. The mechanisms of *Tanshinone IIA* for treating liver fibrosis were predicted using a network pharmacology approach [20].

In this study, network pharmacology was employed to analyze the targets and mechanism of silibinin in the treatment of tumors. The predicted targets were verified using a Western blot method on adenoid cystic carcinoma (ACC2) cells and Balb/c nude mice. This is the first time that the tumor treatment mechanism of silibinin has been studied through network pharmacology and experimental verification methods. The results might provide a research foundation for the improved application of silibinin in tumor treatment.

## 2. Results

### 2.1. Network Pharmacology Analysis

#### 2.1.1. Target Proteins of Silibinin

A total of 347 targets interacting with silibinin (Table S1) were collected from the PharmMapper (Version 2017), SEA and STITCH databases. Seventy-five targets related to the tumor growth process for silibinin were filtrated by TTD and CTD databases and the data was combined with that from existing literature reports (Table 1).

#### 2.1.2. GO Enrichment Analysis for Targets

**Figure 1:**
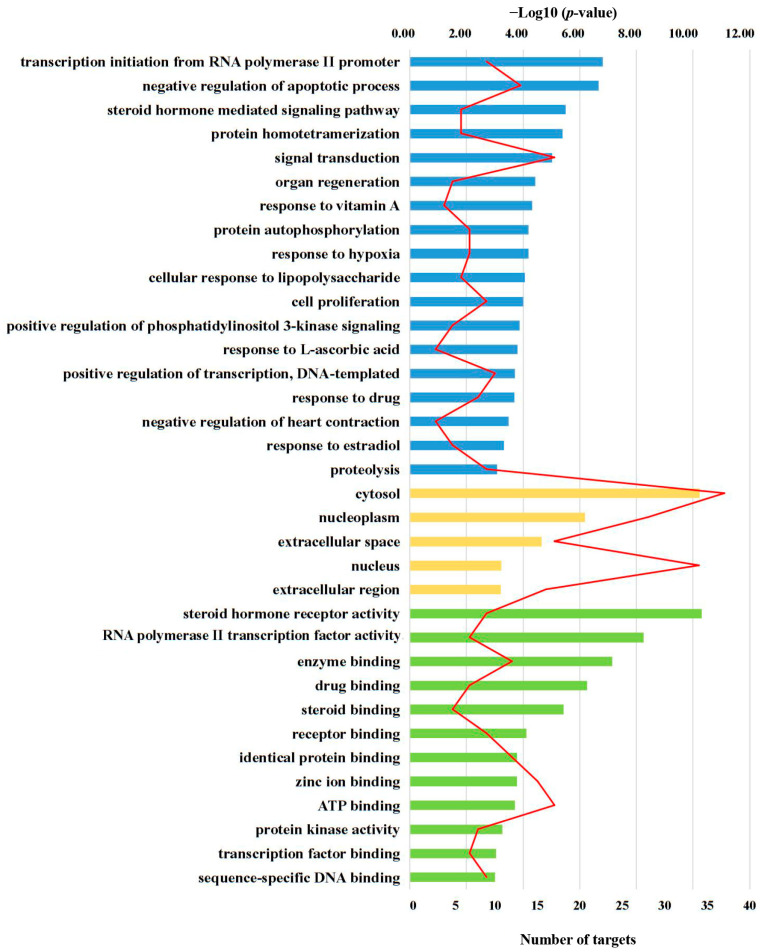
GO enrichment analysis of the identified targets in terms of biological process (blue), cellular components (yellow) and molecular function (green). The order of importance in each term was ranked by −Log10 (*p*-value) with bar chart. The red line chart shows the number of targets in each term.

In order to gain a comprehensive understanding of the functions of these targets, we used DAVID online analysis to conduct a functional enrichment analysis of the target genes [21,22]. Herein, GO terms (*p* < 0.001) showed that the targets were mainly associated with transcription initiation from an RNA polymerase II promoter, and the negative regulation of apoptosis (in biological process, BP), cytosol and nucleoplasm (in cellular component, CC), as well as steroid hormone receptor activity, RNA polymerase II transcription factor activity, ligand-activated sequence-specific DNA binding, enzyme binding, and drug binding (in molecular function, MF) (Figure 1).

#### 2.1.3. Network Construction and Mechanism Analysis

**Figure 2:**
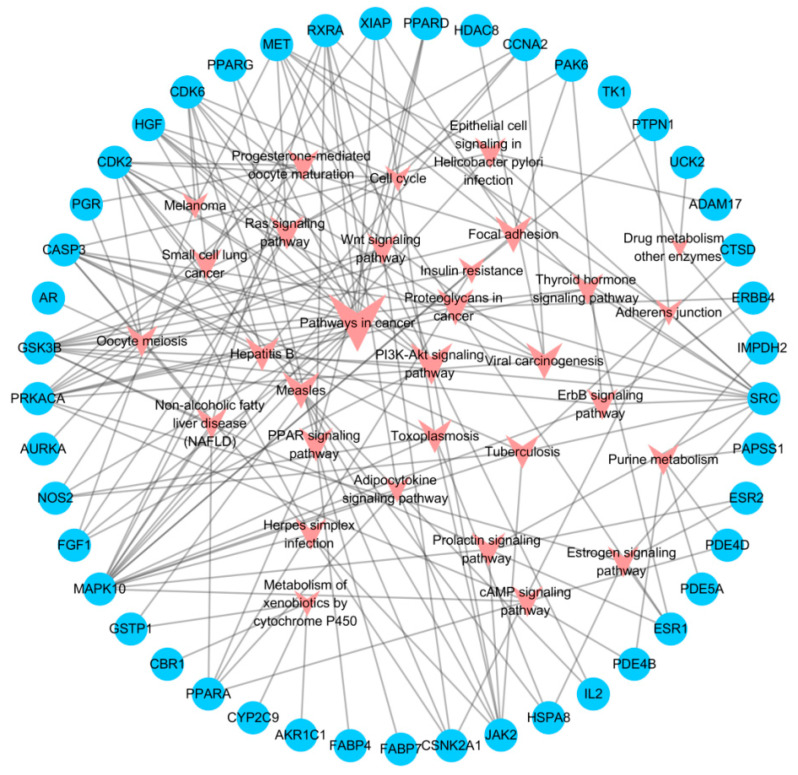
The T-P network of silibinin. The circles are targets, and the arrows represent pathways for which the size is proportional to its degree.

The selected target protein information was imported into the DAVID database to obtain the pathways of target enrichment. The T-P network was constructed using Cytoscape software to visualize the corresponding relationship between targets and pathways (Figure 2). The main pathways in the T-P network included cancer pathways (degree = 16), PI3K-Akt signaling pathway (degree = 9), proteoglycans in cancer (degree = 7), measles (degree = 7), focal adhesion (degree = 7) and viral carcinogenesis (degree = 7). The main targets were MAPK10 (degree = 16), GSK3B (degree = 11), SRC (degree = 11), CDK2 (degree = 10), PRKACA (degree = 10) and CASP3 (degree = 9).

**Figure 3:**
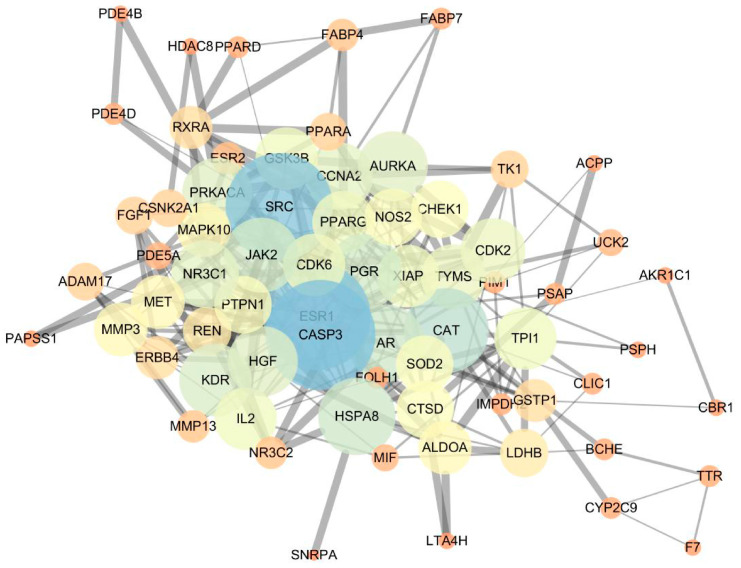
PPI network of intersection targets.

To further investigate the significance of the selected targets, the PPI of the aforementioned target proteins was constructed using the String platform, and the PPI network was generated through Cytoscape 3.6.1 (Figure 3). The PPI network consisted of 69 nodes and 303 edges, and a large number of edges indicates stronger protein interactions. CASP3, SRC, ESR1, JAK2, PRKACA, HSPA8 and CAT exhibited stronger interactions with other factors, suggesting that they may be the key targets for tumor treatment.

### 2.2. Silibinin Inhibited ACC2 Cell Viability and Proliferation

**Figure 4:**
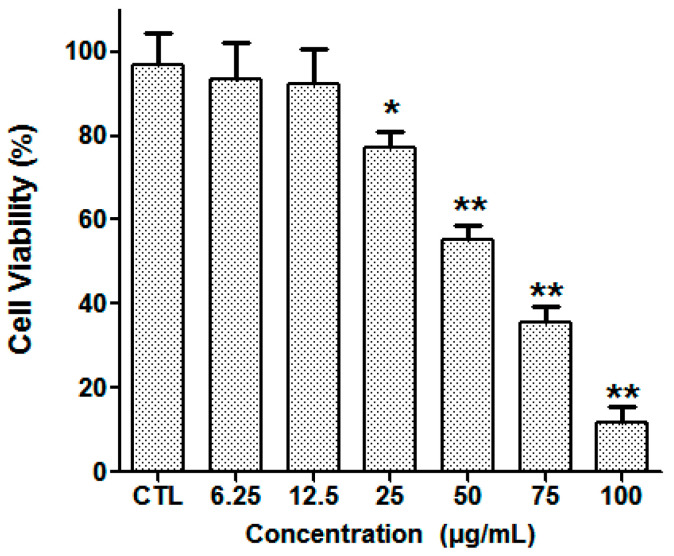
The ACC2 cell viability after treatment with silibinin. * *p* < 0.05, ** *p* < 0.01 vs. CTL, CTL: normal control group.

The cytotoxicity of silibinin against ACC2 cells was investigated using the MTT assay. When ACC2 cells were incubated with different concentrations of silibinin for 24 h, a dose-dependent decrease in cell viability was observed (Figure 4). The IC_50_ value (concentration that inhibits cell growth by 50%) was 59.54 ± 2.32 μg/mL.

**Figure 5:**
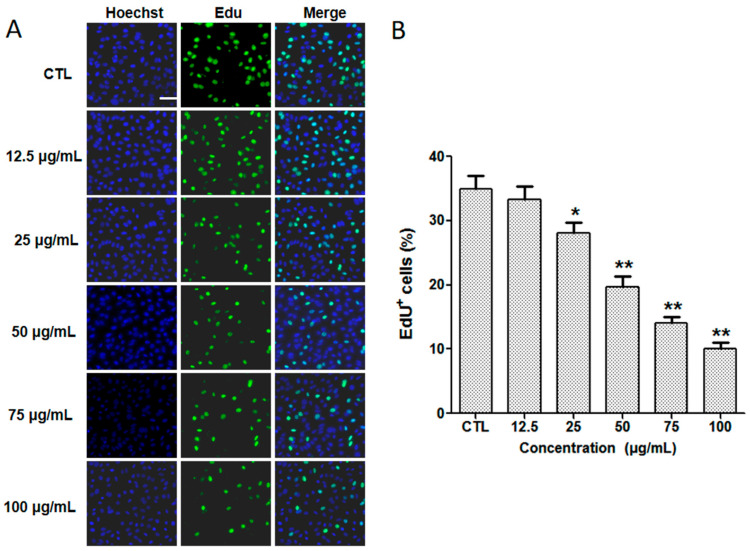
(**A**) Cells stained with Hoechst 33342 and EdU dye after 24 h of incubation with different concentrations of silibinin. Scale bar = 100 μm. (**B**) Percentage of EdU-positive cells after treatment with different concentrations of silibinin. * *p* < 0.05, ** *p* < 0.01 vs. CTL, CTL: normal control group.

The cell proliferation effect of silibinin was examined using cell dual staining with Hoechst 33342 and EdU dye and visualized by a fluorescence microscope. Hoechst 33342 is a cell-penetrant fluorescent dye, and is used to label the DNA of healthy cells [23]. EdU is an alkynylsubstituted thymidine analogue that specifically labels the replicating DNA of cells [24]. The cells in the normal control group showed strong green fluorescence after incubation with EdU for 2 h (Figure 5). The fluorescence was decreased in a dose-dependent manner in the silibinin groups. Based on the above results, it could be concluded that silibinin exhibited the inhibition effects of cell viability and proliferation.

### 2.3. Detection of Apoptosis by Hoechst 33342/PI Staining In Vitro

**Figure 6:**
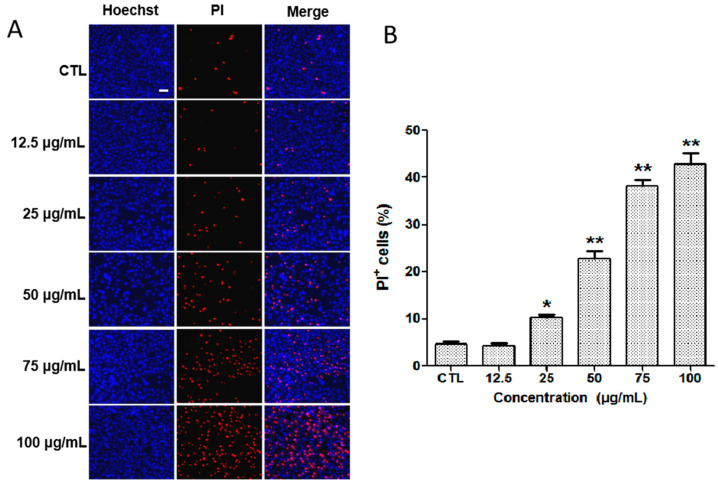
(**A**) Cells stained with Hoechst 33342 and PI dye after 24 h of incubation with different concentrations of silibinin. Scale bar = 100 μm. (**B**) Percentage of PI-positive cells after treatment with different concentrations of silibinin. * *p* < 0.05, ** *p* < 0.01 vs. CTL, CTL: normal control group.

The apoptosis of ACC2 cells treated with different concentrations of silibinin was investigated using the Hoechst 33342/PI double-staining strategy. Hoechst 33342 is used to label the DNA of healthy cells [23], whereas, the PI can bind with DNA in apoptosis cells, producing red fluorescence [25]. The cells in the normal control group (CTL) showed strong blue fluorescence stained by Hoechst 33342 dye and weak red fluorescence produced in a small amount of death cells stained with PI dye (Figure 6). After treatment with different concentrations of silibinin, there was an obvious enhancement of red fluorescence signal in a dose-dependent manner. The above results indicate that silibinin could induce the apoptosis of ACC2 cells.

### 2.4. Cell Migration Effect of Silibinin In Vitro

**Figure 7:**
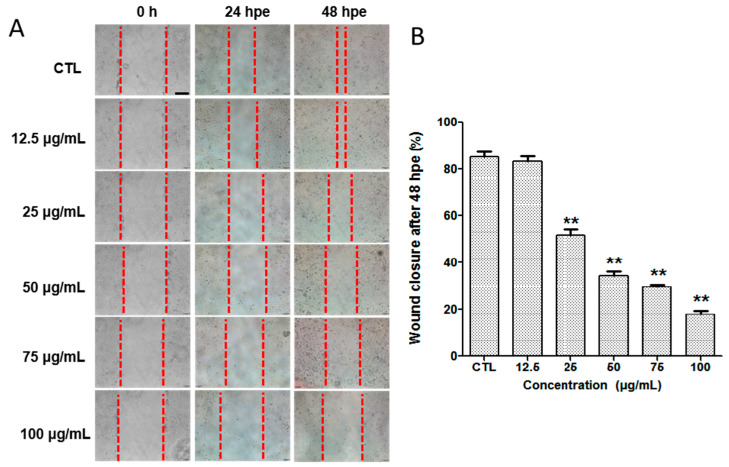
(**A**) The pictures of ACC2 cell migration in wound closure assay at 0, 24 and 48 h. The red dashed lines indicate the boundary of the wound. (**B**) The rate of wound closure of ACC2 cells treated with silibinin at 48 h. ** *p* < 0.01 vs. CTL, CTL: normal control group.

The ability of silibinin to inhibit ACC2 cell migration was examined by the wound closure assay described previously [26,27]. The cells were treated with various concentrations of silibinin for 48 h after scraping using a pipette tip. The migration distance was photographed every 24 h. In the normal control group, the wound had almost closed after 48 h, whereas treatment with silibinin resulted in a significantly larger wound area (Figure 7). The results indicate that silibinin displayed an excellent ability to inhibit ACC2 cell migration.

### 2.5. Effect of Silibinin on Tumor-Related Targets In Vitro

**Figure 8:**
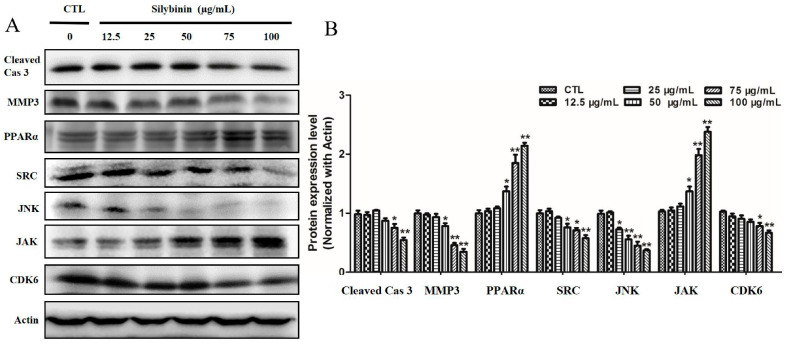
(**A**) Expression of studied proteins in ACC2 cells after treatment with silibinin analyzed by Western blot analysis. Cas 3: CASP3. (**B**) Relative expression of studied proteins to β-actin. * *p* < 0.05, ** *p* < 0.01 vs. CTL, CTL: normal control group.

The network pharmacology results showed that cleaved CASP3, SRC, MMP3, PPARα, JAK, JNK and CDK6 are potential targets of silibinin for the treatment of tumors. In order to verify whether these targets played a role in the treatment of tumors via silibinin as we expected, the expression levels of these proteins were detected in vitro using Western blot analysis. As shown in Figure 8, treatment of cells with silibinin significantly reduced the expression of cleaved CASP3, MMP3, SRC, JNK and CDK6 and increased the expression of PPARα and JAK.

### 2.6. Effect of Silibinin on Tumor-Bearing Mice

**Figure 9:**
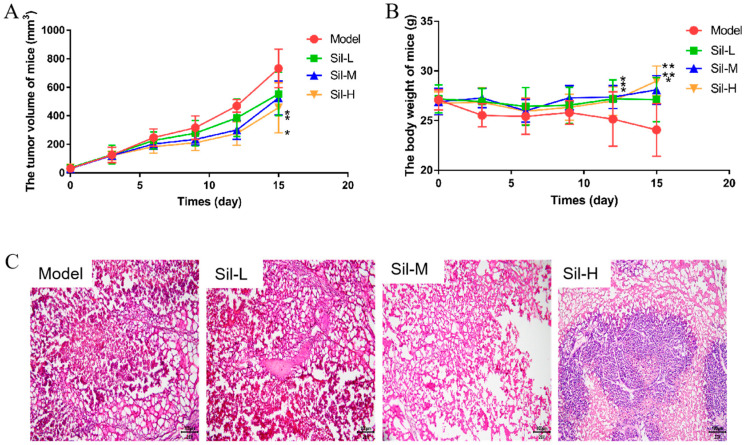
Effect of Silibinin on Tumor-Bearing Mice. (**A**) The tumor volume, (**B**) body weight, (**C**) histopathological sections of tumor. The scale bar is 100 μm. * *p* < 0.05, ** *p* < 0.01 vs. model group.

In this experiment, the body weight of the mice and the tumor volume were recorded to additionally illustrate the effect of silibinin on tumor-bearing mice (Figure 9A,B). The tumor volume of all mice showed an increasing trend, but compared with that of the model control group, the volume of the administration group increased more slowly, especially in the high-dose group. The body weight of all mice was increased during administration. However, the weight of mice in the model control group was lower than that of mice in other groups.

Histopathological sections can visibly assess the effect of silibinin on tumor-bearing mice. The result of H&E staining of tumor tissues showed that the salivary gland structure of the model group was disordered, and the overall staining of the nucleus and cell membrane was poor (Figure 9C). There are many cancer cells, which are large in size, rich in cytoplasm and display a disordered and compact cell arrangement. The cell volume shrunk obviously, and the nucleolus and nuclear membrane were not clear, which affected the interpretation of the slice. After silibinin treatment, the salivary gland structure of the tissue was clearer, the cells were fewer in number, the integrity of the nucleus and cell membrane was relatively good, the nucleolus was small, the cell density was loose, and some nuclei and cytoplasm showed pyknotic necrosis-like changes.

### 2.7. Effect of Silibinin on Tumor-Related Targets In Vivo

**Figure 10:**
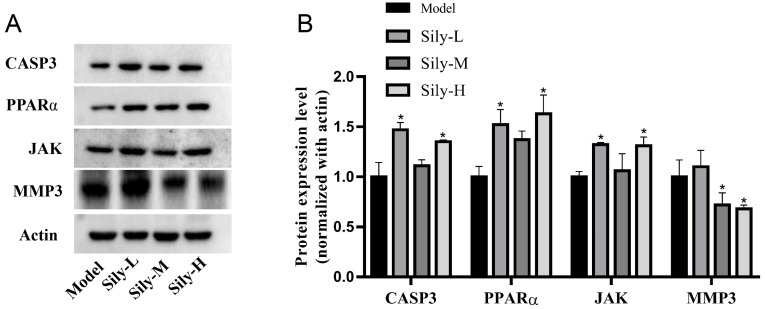
(**A**) Expression of studied proteins in mice after treatment with silibinin, analyzed by Western blot analysis. (**B**) Relative expression of studied proteins to β-actin. * *p* < 0.05 vs. Model.

Silibinin could decrease the tumor volume and delay the tumor tissue development. Moreover, the in vitro Western blot results indicate that the possible molecular mechanism involved inhibiting cleaved CASP3, MMP3, SRC, MAPK10 and CDK6, and activating PPARα, JAK. In order to verify whether these targets played a role in the silibinin-based treatment of tumors in mice, the expression levels of CASP3, MMP3, PPARα and JAK were detected in vivo using Western blot analysis (Figure 10). The results indicate that the expression of CASP3, PPARα and JAK was increased and that of MMP3 was reduced. These results are consistent with the Western blot results of the cell experiments.

## 3. Material and Methods

### 3.1. Network Pharmacology Analysis

#### 3.1.1. Target Fishing

To identify potential targets of silibinin, its sdf format was uploaded to the databases of PharmMapper (http://www.lilab-ecust.cn/pharmmapper/index.html, accessed on 25 May 2022) [28], Similarity Ensemble Approach (SEA, http://sea.bkslab.org/, accessed on 25 May 2022) [29] and STITCH (http://stitch.embl.de/, accessed on 25 May 2022) [30]. The potential targets were filtrated using the Therapeutic Target Database (TTD, https://idrblab.net/ttd/, accessed on 25 May 2022) and Comparative Toxicogenomics Database (CTD, http://ctdbase.org/, accessed on 25 May 2022) (screening species was “Homo sapiens”) in combination with existing literature reports to obtain targets related to the tumor process. The selected target proteins were utilized to construct a protein–protein interaction (PPI) network model on the String (https://string-db.org/, accessed on 25 May 2022) platform [31].

#### 3.1.2. KEGG Pathway and Gene Ontology (GO) Terms Analysis

The selected target protein information was imported into the Database for Annotation, Visualization and Integrated Discovery (DAVID, https://david.ncifcrf.gov/, accessed on 26 May 2022) [21] to obtain information on KEGG pathways and GO terms of the target enrichment. OFFICIAL GENE SYMBOL and Homo Sapiens were selected as the background. *p* ˂ 0.05 was the screening condition for KEGG pathways and GO terms, which excluded a wide range of pathways and GO terms.

#### 3.1.3. Network Construction and Analysis

Data for the correlation between silibinin and selected targets and selected targets and pathways obtained through screening, were integrated into the excel tables. Subsequently, target–pathway (T-P) networks were constructed by Cytoscape 3.6.1 software to visualize the data. The PPI of selected target proteins was established using the String (https://string-db.org/, accessed on 28 May 2022) platform, and the PPI network was then generated by Cytoscape 3.6.1.

### 3.2. Chemicals and Reagents

Silibinin (purity ≥ 99.0%) and hematoxylin and eosin (H&E) were procured from Beijing Solarbio Science & Technology Co., Ltd. (Beijing, China). Other solvents and reagents were obtained as analytical grade and used without further purification. 3-(4,5-Dimethylthiazol-2-yl)-2,5-diphenyltetrazolium bromide (MTT) was purchased from Sigma (Tokyo, Japan). Hoechst 33342/propidium iodide (PI) double staining kit was bought from Genview (Beijing, China), and a 5-ethynyl-20-deoxyuridine (EdU) assay kit was acquired from Ribobio (Guangzhou, China). Antibodies including Anti-CASP3, Anti-MMP3, Anti-SRC, Anti-MAPK10, Anti-CDK6, Anti-PPARα and Anti-JAK were obtained from Cell Signaling Technology (Shanghai, China).

### 3.3. Cell Culture and Treatments

ACC2 cells were procured from the cell bank of the Shanghai Institute for Biological Sciences, Chinese Academy of Sciences (Shanghai, China). The cells were cultured in Dulbecco’s Modified Eagle Medium (DMEM), supplemented with 10% fetal bovine serum (FBS), 100 U/mL of penicillin and 100 mg/mL of streptomycin (Thermo Fisher Scientific, Waltham, MA, USA) in a 5% CO_2_ incubator at 37 °C. Cell suspensions with 1 × 10^4^ cells/mL of 100 μL were added to a 96-well plate. The groups included a normal control group, and silibinin groups (6.25, 12.5, 25, 50, 75 and 100 μg/mL). After incubation for 24 h, the medium in the test wells were replaced with DMEM medium containing 0.5% DMSO, and six concentrations of silibinin (6.25, 12.5, 25, 50, 75 and 100 μg/mL). The wells containing 0.1% DMSO were set as a normal control group. Five replicates were set for each group. Then, ACC2 cells were collected and lysed using RIPA buffer.

### 3.4. MTT Assay

The cell viability was assessed using the MTT method [32]. After incubation with test compounds for 24 h, MTT (5 mg/mL, 10 μL) was added to each well. Following an additional 4 h incubation, DMSO (200 μL) was added. The optical density (OD) values were measured at 560 nm using a microplate reader. All experiments were conducted in triplicate.

### 3.5. Cell Proliferation

The cell proliferation of silibinin was assessed using the EdU assay kit (Ribobio), as previously described [33]. In brief, 100 μL cell suspensions containing 5 × 10^4^ cells/well were seeded into a 96-well glass plate and incubated overnight. Then, the cells were exposed to test compounds for 24 h. The cell culture medium was replaced with fresh medium containing 50 μM EdU and incubated for an additional 2 h. Then, the cells were fixed with 4% formaldehyde for 20 min, permeated with 0.5% Triton X-100 for 10 min, cultured with Apollo reaction mixture for 30 min, and stained with Hoechst 33342 for 10 min. The cell images were obtained using a fluorescence microscope and analyzed using ImageJ software (version 1.53 src). The proliferation rate was determined as the ratio of EdU-positive cells to Hoechst 33342-positive cells in each field.

### 3.6. Cell Apoptosis

The pro-apoptotic effects of silibinin on ACC2 cells were assessed using Hoechst 33342/PI double staining, as previously described [33]. The cell suspensions with 5000 cells/well were placed in 96-well plates and incubated for 24 h. The cells were administrated with silibinin (12.5, 25, 50, 75 and 100 μg/mL) for 24 h. The apoptotic cells were detected using fluorescence microscopy, after double staining with Hoechst 33342/PI for 15 min in the dark at 37 °C. The apoptotic cells were identified based on the positive staining of PI and Hoechst 33342, as well as the absence of nuclear fragmentation. The apoptosis rate was determined by the ratio of PI-positive cells to Hoechst 33342-positive cells in each field. The experiments were conducted in triplicate.

### 3.7. Wound Closure Assay

The ACC2 migration assay was conducted using the wound-healing method, as previously described [26,27,33]. The cells of 3 × 10^5^ cells/well were cultured in 24-well plates for 24 h, and horizontally scraped using a sterile pipette tip. The cells were subsequently washed twice using phosphate-buffered saline. Then, fresh medium containing 10% FBS and silibinin (12.5, 25, 50, 75 and 100 μg/mL) was added. Images were captured at 0, 24 and 48 h after wounding and analyzed with Image-Pro Plus software (version 6.0). The experiments were performed in triplicate.

### 3.8. Animals and Treatment

Twenty-eight male Balb/c nude mice at four weeks were obtained from GemPharmatech Co., Ltd. (Nanjing, China. License number: SCXK(Su)2018-0008). The experiment was conducted according to the standard ethical guidelines that were approved by the Ethics Committee of the Jiangxi Zhonghong Boyuan Biotech Co., Ltd. (Nanchang, China, 2022040101). All mice were bred at 20~26 °C and 40~70% humidity under a 12 h light/dark cycle with enough food and water. After a week of acclimation, the mice were inoculated with ACC-2 cells for tumor formation, and divided into four groups (*n* = 7): a model control group (inoculated with ACC-2 cells) and three silibinin treatment groups: a high-dosage silibinin group (Sily-H), medium-dosage silibinin group (Sily-M) and low-dosage silibinin group (Sily-L). After tumor formation, the mice in the model control group were administrated with brine. The mice in the Sily-H, Sily-M and Sily-L groups were given silibinin at dosages of 250, 125, 62.5 mg/kg/d, respectively, by intraperitoneal injection for two weeks. All mice were fasted immediately after modeling, provided with adequate water simultaneously, and euthanized after 8 h. The tumor tissues were excised for histopathological and Western blot analyses, as well as protein extraction. All mice were fasted immediately after the modeling process and after 8 h.

### 3.9. Histopathological Analysis

The histopathological analysis was performed as in our previous study [34]. The tumor tissue sections were fixed with fixative for 24 h, embedded in paraffin and sliced into 4 μm thick sections. The paraffin sections were dewaxed, and then stained with H&E. The changes to cells and tumor sections were recorded by microscope (CX41, Olympus, Tokyo, Japan).

### 3.10. Western Blot Analysis

Protein concentrations of ACC-2 cells and tumor tissues were quantified by the BCA protein assay kit (Beyotime, Shanghai, China). An equal amount of protein in each group was separated using SDS-PAGE gel electrophoresis and transferred onto a nitrocellulose membrane. After being blocked with 5% BSA for 1 h, the membranes were incubated overnight at 4 °C with primary antibodies (Anti-CASP3, Anti-MMP3, Anti-SRC, Anti-MAPK10, Anti-CDK6, Anti-PPARα and Anti-JAK). The membranes were then washed three times with tris-buffered saline + 0.1% Tween 20, and incubated for 1 h with the corresponding secondary antibodies at 37 °C. The visualization of the protein bands was performed with an ECL advanced Western blotting detection kit.

### 3.11. Statistical Analysis

Statistical analyses were performed using GraphPad Prism 7.0, and data were presented as mean ± standard deviation (SD). The *t*-test was used as a simple comparison test.

## 4. Discussion

Tumors seriously threaten human life, and have become the second leading cause of death in the world after cardiovascular diseases [1]. TCM can prevent and treat tumors effectively with its unique advantages and minimal side effects [3]. *S*. *marianum* (L.) Gaertn, a TCM herb, has been used to treat liver diseases for thousands of years [6]. Silibinin is the active ingredient with multiple activities, including protecting the liver, lowering blood lipid content and anti-oxidation and anti-tumor effects [10]. Silibinin can initiate apoptosis mechanisms (including intracellular and extracellular mechanisms) and induce the death of various cancer cells. It can strongly inhibit DNA synthesis and regulate the level of enzymes related to apoptosis [35,36,37]. It also has a synergistic effect with doxorubicin, cisplatin, carboplatin, etc., and increases the apoptosis rate of tumor cells after combined use [38,39]. Therefore, it has important clinical significance for tumor treatment. However, the molecular anti-tumor mechanism of silibinin is still unclear. In this study, we used network pharmacology and in vitro and in vivo experiments to analyze and verify the potential targets and mechanism of silibinin in the treatment of tumors for the first time. The predicted targets were verified by the Western blot method on ACC2 cells.

Network pharmacology with the relevant databases and software was used to predict potential targets like CASP3, SRC, ESR1 and JAK2 and pathways such as PI3K-Akt, proteoglycans and focal adhesion in cancer. Based on the results of silibinin on ACC2 cells, it could be concluded that silibinin (concentrations from 25 to 100 μg/mL) exhibited the inhibition effects of cell viability, proliferation and migration, as well as the induction effects of cell apoptosis. We found that the treatment of ACC2 cells with silibinin significantly reduced the expression of cleaved CASP3, MMP3, SRC, JNK and CDK6 and increased the expression of PPARα and JAK (Figure 8). This was also confirmed by in vivo Western blot mouse experiments. 

CASP3, which can be initiated by extrinsic (death receptor) and intrinsic (mitochondrial) apoptotic pathways, can cleave a variety of substrates including itself, and then lead to DNA fragmentation, eventually resulting in cell death. Cleaved CASP3 promotes the repopulation of tumors from a small number of surviving cells, and elevated expression levels of cleaved (and thus activated) CASP3 in tumors are associated with poorer treatment outcomes in cancer patients [40]. The SRC family kinases play a role in cell proliferation, differentiation, stress, apoptosis and ECM accumulation [41]. Additionally, they activate numerous downstream signaling pathways such as MAPK, PI3K/Akt and TLR4 [42,43]. MMP3 is involved in the family of MMPs and participates in the process of metastasis with the ability of cleaving various matrix protein substrates such as collagen types, fibronectin, gelatins, proteoglycanase and E-cadherin [44]. MMP3 is over-expressed in various human tumor tissues, and is considered a potential diagnostic or prognostic biomarker of some cancers [45,46,47,48]. PPARα is one of the peroxisome-proliferator-activated receptors (PPARs) [49]. Previous studies have proved that PPARα plays a key role in regulating cell autophagy, metabolic homeostasis and tumorigenesis. PPARα and retinoid X receptor (RXR) bind to specific DNA sequences in the form of heterodimers, thereby stimulating the transcription of multiple target genes [50,51]. JAK is a non-transmembrane tyrosine kinase. The JAK pathway is a classic cell signal-transduction pathway, which plays an important role in regulating the proliferation, migration, differentiation, and cell cycle of normal cells. At the same time, the activation of the JAK pathway is also related to the occurrence, growth, transfer and apoptosis of tumor cells [52]. MAPK10 is a member of the JNK subgroup in the MAPK superfamily and has been proposed as an epigenetically inactive tumor suppressor [53]. CDK promotes the activities of cell life [54]. The decrease in CDK6 expression level indicates that silibinin might cause the G1 phase block of the cell cycle, inducing cell apoptosis.

These results revealed that the mechanisms of silibinin for the treatment of tumors were related to the inhibition of cleaved CASP3, MMP3, SRC, MAPK10, CDK6 and activation of PPARα, JAK, which lead to the inhibition of cell viability, proliferation and migration, as well as cell apoptosis. These proteins are predominantly involved in cancer pathways, the PI3K-Akt signaling pathway and viral carcinogenesis pathways. The findings of the present study further demonstrate that silibinin treated tumors via a combination of multiple targets and pathways. Moreover, these targets may be combined to evaluate the therapeutic effect of silibinin. Our study may contribute to the application of silibinin in clinical tumor therapy and lies a foundation for its development in the future.

## 5. Conclusions

Natural compounds play a huge role in global healthcare and traditional medicine systems. Silibinin has been found to have good anti-tumor activity. Studies have shown that silibinin can participate in different stages of carcinogenesis: proliferation inhibition, cell cycle regulation, cell apoptosis induction, angiogenesis inhibition, migration inhibition, etc. In this study, according to network pharmacology predictions, the ACC2 cells and Balb/c nude mice were used as the research object to explore the anti-tumor effects of silibinin, and validate silibinin’s potential anti-tumor mechanism. Our study demonstrates that silibinin could significantly inhibit cell viability, proliferation and migration, and induce cell apoptosis, thus exerting an anti-tumor effect. The possible molecular mechanism involves inhibiting cleaved CASP3, MMP3, SRC, MAPK10 and CDK6, and activating PPARα and JAK, which are typically involved in cancer pathways, the PI3K-Akt signaling pathway and viral carcinogenesis pathways. Overall, our results suggest that silibinin has the potential to alleviate tumors through its impact on multiple targets and signaling pathways. These results provide a comprehensive understanding of the pharmacological mechanisms of silibinin in tumor treatment.

## Figures and Tables

**Table 1:** Target information of silibinin.

No.	Target	Uniprot ID	Target Name
T1	cGMP-specific 3,5-cyclic phosphodiesterase	T94879	PDE5A
T2	Estrogen receptor	T89534	ESR1
T3	Stromelysin-1	T86702	MMP3
T4	Cholinesterase	T99799	BCHE
T5	Mitogen-activated protein kinase 10	T85421	MAPK10
T6	Estrogen receptor beta	T80896	ESR2
T7	Transthyretin	T86462	TTR
T8	Proto-oncogene serine/threonine-protein kinase Pim-1	T50594	PIM1
T9	U1 small nuclear ribonucleoprotein A	P09012	SNRPA
T10	Glutathione S-transferase P	T21669	GSTP1
T11	Tyrosine-protein phosphatase non-receptor type 1	T89529	PTPN1
T12	Peroxisome proliferator-activated receptor gamma	P37231	PPARG
T13	Cathepsin D	T67102	CTSD
T14	Macrophage migration inhibitory factor	T39977	MIF
T15	Cell division protein kinase 2	P24941	CDK2
T16	Serine/threonine-protein kinase Chk1	T62449	CHK1
T17	Mineralocorticoid receptor	T72168	MR
T18	Leukotriene A-4 hydrolase	T03691	LTA4H
T19	Progesterone receptor	T22939	PGR
T20	Cyclin-A2	T58470	CCNA2
T21	cAMP-specific 3,5-cyclic phosphodiesterase 4B	T10265	PDE4B
T22	Glutamate carboxypeptidase 2	T97071	FOLH1
T23	Collagenase 3	T34296	MMP13
T24	Phosphoserine phosphatase	P78330	PSPH
T25	Peroxisome proliferator-activated receptor delta	Q03181	PPARD
T26	Proto-oncogene tyrosine-protein kinase Src	T85943	SRC
T27	Thymidylate synthase	T98397	TYMS
T28	Androgen receptor	T11211	AR
T29	Cell division protein kinase 6	T89361	CDK6
T30	Aldo-keto reductase family 1 member C1	Q04828	AKR1C1
T31	cAMP-specific 3,5-cyclic phosphodiesterase 4D	T02001	PDE4D
T32	Casein kinase II subunit alpha	P68400	CSNK2A1
T33	Chloride intracellular channel protein 1	O00299	CLIC1
T34	Heparin-binding growth factor 1	T18639	FGF1
T35	Glycogen synthase kinase-3 beta	T70977	GSK3B
T36	cAMP-dependent protein kinase catalytic subunit alpha	P17612	PRKACA
T37	Carbonyl reductase [NADPH] 1	T70518	CBR1
T38	Inosine-5-monophosphate dehydrogenase 2	T89360	IMPDH2
T39	L-lactate dehydrogenase B chain	P07195	LDHB
T40	Heat shock cognate 71 kDa protein	P11142	HSPA8
T41	Superoxide dismutase [Mn], mitochondrial	P04179	SOD2
T42	Retinoic acid receptor RXR-alpha	T13726	RXRA
T43	Caspase-3	T57943	CASP3
T44	Vascular endothelial growth factor receptor 2	P35968	VEGFR2
T45	Coagulation factor VII	T43332	F7
T46	Cytochrome P450 2C9	T19244	CYP2C9
T47	Triosephosphate isomerase	T59130	TPI
T48	Peroxisome proliferator-activated receptor alpha	T86591	PPARα
T49	Catalase	T01597	CAT
T50	Hepatocyte growth factor receptor	T40474	MET
T51	Serine/threonine-protein kinase 6	O14965	AURKA
T52	Prostatic acid phosphatase	T93283	ACPP
T53	Serine/threonine-protein kinase PAK 6	Q9NQU5	PAK6
T54	Disintegrin and metalloproteinase domain-containing protein 17	P78536	ADAM17
T55	Thymidine kinase, cytosolic	P04183	TK1
T56	Protein-glutamine gamma-glutamyltransferase E	Q08188	TGM3
T57	Histone deacetylase 8	T28887	HDAC8
T58	Receptor tyrosine-protein kinase erbB-4	T92057	ELNE
T59	Fructose-bisphosphate aldolase A	P04075	ERBB4
T60	Uridine-cytidine kinase 2	Q9BZX2	ALDOA
T61	Fatty acid-binding protein, adipocyte	T07217	UCK2
T62	Proactivator polypeptide	P07602	FABP4
T63	Tyrosine-protein kinase JAK2	T87554	PSAP
T64	Hepatocyte growth factor	P14210	JAK2
T65	Renin	P00797	HGF
T66	Fatty acid-binding protein, brain	O15540	REN
T67	Bifunctional 3-phosphoadenosine 5-phosphosulfate synthetase 1	O43252	FABP7
T68	Baculoviral IAP repeat-containing protein 4	P98170	PAPSS1
T69	Endoplasmic reticulum mannosyl-oligosaccharide 1,2-alpha-mannosidase	Q9UKM7	XIAP
T70	Nitric oxide synthase, inducible	T02703	MAN1B1
T71	Interleukin-2	T61698	NOS2
T72	Glucocorticoid receptor	P04150	IL2
T73	Angiopoietin-1 receptor	Q02763	NR3C1
T74	NADH dehydrogenase [ubiquinone] 1 alpha subcomplex subunit 4-like 2	Q9NRX3	NDUFA4L2
T75	Prostaglandin D2 receptor	Q13258	PGD

## Data Availability

The data presented in this study are available upon request from the corresponding author.

## Supplementary Materials

The following supporting information can be downloaded at: https://www.mdpi.com/article/10.3390/molecules29081901/s1, Table S1: The targets interacting with silybinin.
