# Supplementary material for: Anti-Tumor Activity and Mechanism of Silibinin Based on Network Pharmacology and Experimental Verification
Source: Molecules. 2024 Apr 22;29(8):1901. doi: 10.3390/molecules29081901 (PMC11054111; doi:10.3390/molecules29081901)
Supplement: Supplementary file 1 [file molecules-29-01901-s001.zip › molecules-2955703-supplementary.pdf]

Anti-Tumor Activity and Mechanism of Silibinin Based on Network Pharmacology and Experimental Verification

Peihai Li [1,†], Dexu Wang [1,†], Xueliang Yang [1], Changyu Liu [1], Xiaobin Li [1], Xuanming Zhang [1], Kechun Liu [1], Yun Zhang [1], Mengqi Zhang [2,*], Changyun Wang [3,*] and Rongchun Wang [1,*]

[1] Engineering Research Center of Zebrafish Models for Human Diseases and Drug Screening of Shandong Province, Biology Institute, Qilu University of Technology (Shandong Academy of Sciences), Jinan 250103, China; liph@sdas.org (P.L.); dexuwangbio@163.com (D.W.)

[2] Key Laboratory of Novel Food Resources Processing, Ministry of Agriculture and Rural Affairs/Key Laboratory of Agro-Products Processing Technology of Shandong Province, Institute of Agro-Food Science and Technology, Shandong Academy of Agricultural Sciences, Jinan 250100, China

[3] Key Laboratory of Marine Drugs, The Ministry of Education of China, School of Medicine and Pharmacy, Ocean University of China, Qingdao 266003, China

* Correspondence: mengqi139@126.com (M.Z.); changyun@ouc.edu.cn (C.W.); 1wangrongchun@163.com (R.W.)

† These authors contributed equally to this work.

Table S1 The targets interacting with silybinin.

No.	Description	Target Name
1	Complement factor B	CFAB
2	cGMP-specific 3,5-cyclic phosphodiesterase	PDE5A
3	Estrogen receptor	ESR1
4	Methionine aminopeptidase 2	AMPM2
5	Mitogen-activated protein kinase 8	MK08
6	Stromelysin-1	MMP3
7	Glucosylceramidase	GLCM
8	Cholinesterase	CHLE
9	Mitogen-activated protein kinase 10	MAPK10
10	Carbonic anhydrase 2	CAH2
11	Estrogen receptor beta	ESR2
12	Transthyretin	TTR
13	Proto-oncogene serine/threonine-protein kinase Pim-1	PIM1
14	Angiogenin	ANGI
15	U1 small nuclear ribonucleoprotein A	SNRPA
16	Glutathione S-transferase P	GSTP1
17	Tyrosine-protein phosphatase non-receptor type 1	PTN1
18	Peroxisome proliferator-activated receptor gamma	PPARG
19	Cathepsin D	CATD
20	Macrophage migration inhibitory factor	MIF
21	Branched-chain-amino-acid aminotransferase, mitochondrial	BCAT2
22	Cell division protein kinase 2	CDK2
23	Serine/threonine-protein kinase Chk1	CHK1
24	Mineralocorticoid receptor	MR
25	Sex hormone-binding globulin	SHBG
26	Leukotriene A-4 hydrolase	LKHA4
27	Progesterone receptor	PRGR
28	Early endosome antigen 1	EEA1
29	3-phosphoinositide-dependent protein kinase 1	PDPK1
30	Cyclin-A2	CCNA2
31	cAMP-specific 3,5-cyclic phosphodiesterase 4B	PDE4B
32	Glutamate carboxypeptidase 2	FOLH1
33	Collagenase 3	MMP13
34	Phosphoserine phosphatase	PSPH
35	ADAM 17	ADA17
36	Peroxisome proliferator-activated receptor delta	PPARD
37	Proto-oncogene tyrosine-protein kinase Src	SRC
38	Thymidylate synthase	TYSY
39	Mitogen-activated protein kinase 14	MAPK14
40	Phospholipase A2, membrane associated	PLA2G2A

41	Androgen receptor	ANDR
42	Carbonic anhydrase 1	CA1
43	Chitotriosidase-1	CHIT1
44	Cell division protein kinase 6	CDK6
45	Glutaredoxin-1	GLRX1
46	Aldo-keto reductase family 1 member C1	AKR1C1
47	Serum albumin	ALBU
48	NAD-dependent malic enzyme, mitochondrial	MAOM
49	Thyroid hormone receptor beta	THRB
50	cAMP-specific 3,5-cyclic phosphodiesterase 4D	PDE4D
51	3-hydroxy-3-methylglutaryl-coenzyme A reductase	HMDH
52	Estradiol 17-beta-dehydrogenase 1	HSD17B1
53	Casein kinase II subunit alpha	CSNK2A1
54	Alcohol dehydrogenase 1C	ADH1C
55	Chloride intracellular channel protein 1	CLIC1
56	Urokinase-type plasminogen activator	UROK
57	Heparin-binding growth factor 1	FGF1
58	Heat shock protein HSP 90-alpha	HS90A
59	Ornithine carbamoyltransferase, mitochondrial	OTC
60	Lanosterol synthase	ERG7
61	Pyruvate dehydrogenase E1 component subunit beta, mitochondrial	PDHB
62	Estrogen-related receptor gamma	ERR3
63	Glycogen synthase kinase-3 beta	GSK3B
64	Serine hydroxymethyltransferase, cytosolic	GLYC
65	cGMP-inhibited 3,5-cyclic phosphodiesterase B	PDE3B
66	Cathepsin K	CATK
67	Corticosteroid 11-beta-dehydrogenase isozyme 1	DHI1
68	Interferon-stimulated gene 20 kDa protein	ISG20
69	Ribosyldihydronicotinamide dehydrogenase [quinone]	NQO2
70	cAMP-dependent protein kinase catalytic subunit alpha	PRKACA
71	Phenylalanine-4-hydroxylase	PH4H
72	Lactoylglutathione lyase	GLO1
73	Carbonyl reductase [NADPH] 1	CBR1
74	B-Raf proto-oncogene serine/threonine-protein kinase	BRAF1
75	UDP-glucose 4-epimerase	GALE
76	Inosine-5-monophosphate dehydrogenase 2	IMDH2
77	Glutathione S-transferase theta-2	GSTT2
78	Coagulation factor X	FA10
79	L-lactate dehydrogenase B chain	LDHB
80	Heat shock cognate 71 kDa protein	HSPA8
81	Superoxide dismutase [Mn], mitochondrial	SOD2
82	Alcohol dehydrogenase class-3	ADHX
83	Cystathionine beta-synthase	CBS

84	Phosphoenolpyruvate carboxykinase, cytosolic [GTP]	PCK1
85	Aldose reductase	ALDR
86	Uridine 5-monophosphate synthase	UMPS
87	Retinoic acid receptor RXR-alpha	RXRA
88	Caspase-3	CASP3
89	Vascular endothelial growth factor receptor 2	VEGFR2
90	Aldo-keto reductase family 1 member C3	AK1C3
91	Ribonuclease 4	RNASE4
92	Flavin reductase	BLVRB
93	Epidermal growth factor receptor	EGFR
94	Bile salt sulfotransferase	SULT2A1
95	Peptidyl-prolyl cis-trans isomerase FKBP1A	FKB1A
96	Coagulation factor VII	F7
97	ADP-ribosyl cyclase 2	BST1
98	Dipeptidyl peptidase 4	DPP4
99	Cytochrome P450 2C9	CYP2C9
100	Triosephosphate isomerase	TPI
101	Peroxisome proliferator-activated receptor alpha	PPARA
102	T-cell surface glycoprotein CD1a	CD1A
103	Catalase	CATA
104	Hepatocyte growth factor receptor	MET
105	Serine/threonine-protein kinase 6	AURKA
106	Prostatic acid phosphatase	PPAP
107	Serine/threonine-protein kinase PAK 6	PAK6
108	Disintegrin and metalloproteinase domain-containing protein 17	ADAM17
109	Thymidine kinase, cytosolic	TK1
110	Estrogen sulfotransferase	ST1E1
111	Glutathione reductase, mitochondrial	GSR
112	Eosinophil cationic protein	RNASE3
113	Protein-glutamine gamma-glutamyltransferase E	TGM3
114	Adenosylhomocysteinase	SAHH
115	Histone deacetylase 8	HDAC8
116	Leukocyte elastase	ELNE
117	L-serine dehydratase	SDSL
118	NAD(P) transhydrogenase, mitochondrial	NNT
119	Bactericidal permeability-increasing protein	BPI
120	Receptor tyrosine-protein kinase erbB-4	ERBB4
121	[Pyruvate dehydrogenase [lipoamide]] kinase isozyme 2, mitochondrial	PDK2
122	Nicotinamide mononucleotide adenylyltransferase 1	NMNAT1
123	Oxysterols receptor LXR-beta	NR1H2
124	Dihydrofolate reductase	DYR
125	Fructose-bisphosphate aldolase A	ALDOA

126	Uridine-cytidine kinase 2	UCK2
127	Fatty acid-binding protein, adipocyte	FABP4
128	Galactosylgalactosylxylosylprotein 3-beta-glucuronosyltransferase 1	B3GA1
129	Arylsulfatase A	ARSA
130	Insulin receptor	INSR
131	Angiotensin-converting enzyme	ACE
132	Dihydroorotate dehydrogenase, mitochondrial	PYRD
133	Proactivator polypeptide	PSAP
134	Pleckstrin homology domain-containing family A member 4	PLEKHA4
135	Fatty acid-binding protein, heart	FABPH
136	Trafficking protein particle complex subunit 3	TRAPPC3
137	Bile acid receptor	NR1H4
138	Gastrotropin	FABP6
139	Pyruvate kinase isozymes R/L	KPYR
140	Hydroxyacylglutathione hydrolase, mitochondrial	HAGH
141	Phosphatidylinositol transfer protein alpha isoform	PITPNA
142	Tyrosine-protein kinase JAK2	JAK2
143	Hepatocyte growth factor	HGF
144	Renin	RENI
145	Fatty acid-binding protein, brain	FABP7
146	Proto-oncogene tyrosine-protein kinase LCK	LCK
147	Bifunctional 3-phosphoadenosine 5-phosphosulfate synthetase 1	PAPSS1
148	Glucose-6-phosphate isomerase	G6PI
149	Baculoviral IAP repeat-containing protein 4	XIAP
150	Sulfotransferase family cytosolic 2B member 1	SULT2B1
151	Bifunctional purine biosynthesis protein PURH	PUR9
152	Dual specificity protein kinase CLK1	CLK1
153	Glutathione-requiring prostaglandin D synthase	PTGD2
154	Deoxycytidine kinase	DCK
155	Nuclear receptor subfamily 1 group I member 2	NR1I2
156	Endoplasmic reticulum mannosyl-oligosaccharide 1,2-alpha-mannosidase	MAN1B1
157	Nicotinamide mononucleotide adenylyltransferase 3	NMNAT3
158	Ornithine aminotransferase, mitochondrial	OAT
159	S-methyl-5-thioadenosine phosphorylase	MTAP
160	RAC-beta serine/threonine-protein kinase	AKT2
161	Hepatocyte nuclear factor 4-gamma	HNF4G
162	Matrix metalloproteinase-9	MMP9
163	Tyrosine-protein kinase HCK	HCK
164	Complement C1s subcomponent	C1S
165	Cellular retinoic acid-binding protein 2	CRABP2
166	Aldo-keto reductase family 1 member C2	AK1C2
167	Nitric oxide synthase, inducible	NOS2
168	Tyrosine-protein kinase ITK/TSK	ITK

169	Glycogen phosphorylase, liver form	PYGL
170	Basic fibroblast growth factor receptor 1	FGFR1
171	Interleukin-2	IL2
172	C-C motif chemokine 5	CCL5
173	Glutathione S-transferase A1	GSTA1
174	Tyrosine-protein kinase JAK3	JAK3
175	Serine--pyruvate aminotransferase	SPYA
176	5(3)-deoxyribonucleotidase, mitochondrial	NT5M
177	Dipeptidase 1	DPEP1
178	Neprilysin	NEP
179	Platelet glycoprotein Ib alpha chain	GP1BA
180	Thymidylate kinase	KTHY
181	Beta-secretase 1	BACE1
182	ADP-ribosylation factor 4	ARF4
183	Glucocorticoid receptor	GCR
184	Glutathione S-transferase A3	GSTA3
185	Angiopoietin-1 receptor	TIE2
186	Betaine--homocysteine S-methyltransferase 1	BHMT
187	Ferrochelatase, mitochondrial	FECH
188	Neutrophil collagenase	MMP8
189	Glutathione S-transferase Mu 1	GSTM1
190	Serine/threonine-protein phosphatase PP1-gamma catalytic subunit	PPP1CC
191	Coagulation factor XI	FA11
192	Hydroxyacyl-coenzyme A dehydrogenase, mitochondrial	HADH
193	Acetyl-CoA acetyltransferase, mitochondrial	ACAT1
194	Chymase	CMA1
195	Vitamin D3 receptor	VDR
196	Adenosine kinase	ADK
197	Cathepsin F	CTSF
198	Cathepsin S	CATS
199	Cell division protein kinase 7	CDK7
200	Farnesyl pyrophosphate synthetase	FPPS
201	Phosphopantothenoylcysteine decarboxylase	PPCDC
202	CD209 antigen	CD209
203	Heat shock 70 kDa protein 1	HSPA1
204	Retinoic acid receptor alpha	RARA
205	Macrophage metalloelastase	MMP12
206	TGF-beta receptor type-1	TGFR1
207	Histo-blood group ABO system transferase	ABO
208	Peptidyl-prolyl cis-trans isomerase FKBP1B	FKB1B
209	GTP-binding protein Rheb	RHEB
210	Lysozyme C	LYZ
211	Cell division control protein 42 homolog	CDC42

212	Glutathione S-transferase omega-1	GSTO1
213	Retinoic acid receptor beta	RARB
214	Neutrophil gelatinase-associated lipocalin	NGAL
215	Signal transducer and activator of transcription 1-alpha/beta	STAT1
216	Growth factor receptor-bound protein 2	GRB2
217	Eukaryotic translation initiation factor 4E	EIF4E
218	MAP kinase-activated protein kinase 2	MAPKAPK2
219	Transforming growth factor beta-2	TGFB2
220	Non-secretory ribonuclease	RNASE2
221	Bifunctional heparan sulfate N-deacetylase/N-sulfotransferase 1	NDST1
222	E-selectin	LYAM2
223	Ras-related protein Rab-5A	RAB5A
224	Rho-related GTP-binding protein RhoE	RND3
225	GTPase HRas	RASH
226	Phosphatidylinositol 3-kinase regulatory subunit alpha	P85A
227	Glutathione S-transferase Mu 2	GSTM2
228	Mast/stem cell growth factor receptor	KIT
229	Tryptophanyl-tRNA synthetase, cytoplasmic	trpS
230	Mitogen-activated protein kinase 12	MK12
231	Dual specificity mitogen-activated protein kinase kinase 1	MP2K1
232	PMS1 protein homolog 2	PMS2
233	RAF proto-oncogene serine/threonine-protein kinase	RAF1
234	Heme oxygenase 1	HMOX1
235	Histidine triad nucleotide-binding protein 1	HINT1
236	Inositol-trisphosphate 3-kinase A	ITPKA
237	ADP-ribosylation factor-like protein 5A	ARL5A
238	Tyrosine-protein kinase ZAP-70	ZAP70
239	Glucose-6-phosphate 1-dehydrogenase	G6PD
240	Ras-related protein Rab-11A	RB11A
241	Ras-related protein Rab-9	RAB9
242	Death-associated protein kinase 1	DAPK1
243	Apoptotic protease-activating factor 1	APAF
244	Riboflavin kinase	RFK
245	Protein kinase C theta type	KPCT
246	ADP-ribosylation factor-like protein 5B	ARL5B
247	Scavenger mRNA-decapping enzyme DcpS	DCPS
248	Phenylethanolamine N-methyltransferase	PNMT
249	Retinoic acid receptor RXR-beta	RXRB
250	Histamine N-methyltransferase	HNMT
251	L-xylulose reductase	DCXR
252	Baculoviral IAP repeat-containing protein 7	BIRC7
253	Spliceosome RNA helicase BAT1	BAT1
254	ADAM 33	ADA33

255	Histone-lysine N-methyltransferase, H3 lysine-79 specific	HLNM
256	Nucleoside diphosphate kinase B	NDKB
257	Tryptase beta-2	TRYB2
258	6-phosphofructo-2-kinase/fructose-2,6-biphosphatase 1	PFKFB1
259	Kinesin-like protein KIF11	KIF11
260	tRNA (cytosine-5-)-methyltransferase	O14717
261	Quinone oxidoreductase	QOR
262	Antigen peptide transporter 1	TAP1
263	S-adenosylmethionine decarboxylase proenzyme	DCAM
264	Deoxyuridine 5-triphosphate nucleotidohydrolase, mitochondrial	DUT
265	Hypoxanthine-guanine phosphoribosyltransferase	HPRT1
266	Sulfotransferase 1A1	ST1A1
267	RAC-alpha serine/threonine-protein kinase	AKT1
268	Tyrosine-protein kinase BTK	BTK
269	GMP reductase 1	GMPR1
270	Caspase-1	CASP1
271	Fibrinogen gamma chain	FIBG
272	Maleylacetoacetate isomerase	GSTZ1
273	GTP-binding nuclear protein Ran	RAN
274	Interstitial collagenase	MMP1
275	UDP-N-acetylhexosamine pyrophosphorylase	UAP1
276	Histone acetyltransferase PCAF	KAT2B
277	Adenylate kinase isoenzyme 1	AK1
278	Ras-related C3 botulinum toxin substrate 1	RAC1
279	FK506-binding protein 3	FKBP3
280	GMP reductase 2	GMPR
281	Ras-related protein Rap-2a	RAP2A
282	Isovaleryl-CoA dehydrogenase, mitochondrial	IVD
283	Nuclear receptor ROR-alpha	RORA
284	Phosphatidylcholine transfer protein	PCTP
285	Amine oxidase [flavin-containing] B	AOFB
286	Pancreatic alpha-amylase	AMYP
287	NAD-dependent deacetylase sirtuin-5	SIRT5
288	Sepiapterin reductase	SPR
289	Carbonic anhydrase 3	CA3
290	Carbonic anhydrase 5B, mitochondrial	CA5B
291	Carbonic anhydrase 13	CA13
292	Carbonic anhydrase 6	CA6
293	Carbonic anhydrase 7	CA7
294	Carbonic anhydrase 4	CA4
295	Carbonic anhydrase 2	CA2
296	Carbonic anhydrase 5A, mitochondrial	CA5A
297	Taste receptor type 2 member 31	TAS2R31

298	Acyl carrier protein, mitochondrial	NDUFAB1
299	Complex I intermediate-associated protein 30, mitochondrial	NDUFAF1
300	NADH dehydrogenase [ubiquinone] 1 alpha subcomplex subunit 1	NDUFA1
301	NADH dehydrogenase [ubiquinone] 1 alpha subcomplex subunit 2	NDUFA2
302	NADH dehydrogenase [ubiquinone] 1 alpha subcomplex subunit 3	NDUFA3
303	NADH dehydrogenase [ubiquinone] 1 alpha subcomplex subunit 4	NDUFA4
304	NADH dehydrogenase [ubiquinone] 1 alpha subcomplex subunit 5	NDUFA5
305	NADH dehydrogenase [ubiquinone] 1 alpha subcomplex subunit 6	NDUFA6
306	NADH dehydrogenase [ubiquinone] 1 alpha subcomplex subunit 7	NDUFA7
307	NADH dehydrogenase [ubiquinone] 1 alpha subcomplex subunit 8	NDUFA8
308	NADH dehydrogenase [ubiquinone] 1 alpha subcomplex subunit 9	NDUFA9
309	NADH dehydrogenase [ubiquinone] 1 alpha subcomplex subunit 10	NDUFA10
310	NADH dehydrogenase [ubiquinone] 1 alpha subcomplex subunit 11	NDUFA11
311	NADH dehydrogenase [ubiquinone] 1 alpha subcomplex subunit 12	NDUFA12
312	NADH dehydrogenase [ubiquinone] 1 alpha subcomplex subunit 13	NDUFA13
313	NADH dehydrogenase [ubiquinone] 1 beta subcomplex subunit 1	NDUFB1
314	NADH dehydrogenase [ubiquinone] 1 beta subcomplex subunit 2	NDUFB2
315	NADH dehydrogenase [ubiquinone] 1 beta subcomplex subunit 3	NDUFB3
316	NADH dehydrogenase [ubiquinone] 1 beta subcomplex subunit 4	NDUFB4
317	NADH dehydrogenase [ubiquinone] 1 beta subcomplex subunit 5	NDUFB5
318	NADH dehydrogenase [ubiquinone] 1 beta subcomplex subunit 6	NDUFB6
319	NADH dehydrogenase [ubiquinone] 1 beta subcomplex subunit 7	NDUFB7
320	NADH dehydrogenase [ubiquinone] 1 beta subcomplex subunit 8	NDUFB8
321	NADH dehydrogenase [ubiquinone] 1 beta subcomplex subunit 9	NDUFB9
322	NADH dehydrogenase [ubiquinone] 1 beta subcomplex subunit 10	NDUFB10
323	NADH dehydrogenase [ubiquinone] 1 beta subcomplex subunit 11	NDUFB11
324	NADH dehydrogenase [ubiquinone] 1 subunit C1, mitochondrial	NDUFC1
325	NADH dehydrogenase [ubiquinone] 1 subunit C2	NDUFC2
327	NADH dehydrogenase [ubiquinone] 1 alpha subcomplex assembly factor 3	NDUFAF3
328	NADH dehydrogenase [ubiquinone] 1 alpha subcomplex assembly factor 4	NDUFAF4
329	NADH-ubiquinone oxidoreductase 75 kDa subunit, mitochondrial	NDUFS1
330	NADH dehydrogenase [ubiquinone] iron-sulfur protein 2, mitochondrial	NDUFS2
331	NADH dehydrogenase [ubiquinone] iron-sulfur protein 3, mitochondrial	NDUFS3
332	NADH dehydrogenase [ubiquinone] iron-sulfur protein 4, mitochondrial	NDUFS4
333	NADH dehydrogenase [ubiquinone] iron-sulfur protein 5	NDUFS5
334	NADH dehydrogenase [ubiquinone] iron-sulfur protein 6, mitochondrial	NDUFS6
335	NADH dehydrogenase [ubiquinone] iron-sulfur protein 7, mitochondrial	NDUFS7
336	NADH dehydrogenase [ubiquinone] iron-sulfur protein 8, mitochondrial	NDUFS8
337	NADH dehydrogenase [ubiquinone] flavoprotein 1, mitochondrial	NDUFV1
338	NADH dehydrogenase [ubiquinone] flavoprotein 2, mitochondrial	NDUFV2
339	NADH dehydrogenase [ubiquinone] flavoprotein 3, mitochondrial	NDUFV3
340	NADH-ubiquinone oxidoreductase chain 1	MT-ND1
341	NADH-ubiquinone oxidoreductase chain 2	MT-ND2

342	NADH-ubiquinone oxidoreductase chain 3	MT-ND3
343	NADH-ubiquinone oxidoreductase chain 4L	MT-ND4L
344	NADH-ubiquinone oxidoreductase chain 5	MT-ND5
345	NADH-ubiquinone oxidoreductase chain 6	MT-ND6
346	NADH dehydrogenase [ubiquinone] 1 alpha subcomplex subunit 4-like 2	NDUFA4L2
347	6-phosphogluconate dehydrogenase, decarboxylating	PGD
